# Defect in cytosolic Neu2 sialidase abrogates lipid metabolism and impairs muscle function in vivo

**DOI:** 10.1038/s41598-022-07033-6

**Published:** 2022-02-25

**Authors:** Mijung Oh, Dae-In Ha, Chaeyeon Son, Jeong Gu Kang, Heeyoun Hwang, Su Bin Moon, Minjeong Kim, Jihae Nam, Jung Soo Kim, Sang Yong Song, Yong-Sam Kim, Sangwoo Park, Jong Shin Yoo, Jeong-Heon Ko, Kyoungsook Park

**Affiliations:** 1 Medical Research Institute, Sungkyunkwan University School of Medicine, Suwon, 16419 Republic of Korea; 2 Genome Editing Research Center, Korea Research Institute of Bioscience and Biotechnology, 34141 Daejeon, Republic of Korea; 3 Research Center for Bioconvergence Analysis, Korea Basic Science Institute, Cheongju, 28119 Republic of Korea; 4 Department of Pathology and Translational Genomics, Samsung Medical Center, Sungkyunkwan University School of Medicine, Seoul, 06351 Republic of Korea; 5 Department of Bio-Molecular Science, KRIBB School of Bioscience, Korea University of Science and Technology (UST), Daejeon, Republic of Korea

**Keywords:** Biochemistry, Biological techniques

## Abstract

Sialic acid (SA) is present in glycoconjugates and important in cell–cell recognition, cell adhesion, and cell growth and as a receptor. Among the four mammalian sialidases, cytosolic NEU2 has a pivotal role in muscle and neuronal differentiation in vitro. However, its biological functions in vivo remain unclear due to its very low expression in humans. However, the presence of cytoplasmic glycoproteins, gangliosides, and lectins involved in cellular metabolism and glycan recognition has suggested the functional importance of cytosolic Neu2 sialidases. We generated a Neu2 knockout mouse model via CRISPR/Cas9-mediated genome engineering and analyzed the offspring littermates at different ages to investigate the in vivo function of cytosolic Neu2 sialidase. Surprisingly, knocking out the *Neu2* gene in vivo abrogated overall lipid metabolism, impairing motor function and leading to diabetes. Consistent with these results, Neu2 knockout led to alterations in sialylated glycoproteins involved in lipid metabolism and muscle function, as shown by glycoproteomics analysis.

## Introduction

Sialic acid (SA) is a group of terminal acidic monosaccharides that modulate the chemical and biological features of glycoconjugates^1^. These SAs modulate various biological processes through changes in conformation and alterations of binding sites of functional molecules in mammals, and they are mainly regulated by sialyltransferases (ST) and sialidases^2,3^. Sialidases exist widely in vertebrates and have important biological roles in cellular functions including cell differentiation, cell growth, and apoptosis^4^. Their comparative endogenous levels vary in tissue- and species-specific manners^5^. Four types of mammalian sialidase (NEU1, NEU2, NEU3, and NEU4) have been identified, and they are encoded by different genes and differ in major subcellular localization and enzymatic properties including substrate specificity^6^.

Cytosolic NEU2 sialidase desialylates a wide range of glycoproteins and gangliosides at near neutral pH, and investigations of the roles of the *Neu2* gene in muscle and neuronal cultured cells have suggested that NEU2 sialidase is a determining factor for muscle and neuronal differentiation in vitro^1^. Unlike those of other sialidases, the biological functions of NEU2 sialidase in vivo remain unclear due to both its lack of involvement in major glycosylation pathways in the ER and Golgi complex and its very low expression level in humans. However, the presence of cytoplasmic glycoproteins, oligosaccharides, gangliosides, and lectins involved in cellular metabolism and recognition of glycan molecules^7^ indicates the functional importance of cytosolic sialidases.

We generated a Neu2 knockout (KO) mouse model via CRISPR/Cas9-mediated genome engineering and analyzed the offspring littermates at different ages to investigate the in vivo function of cytosolic Neu2 sialidase. Surprisingly, our results demonstrate that the most prominent in vivo function of cytosolic Neu2 sialidase is regulation of lipid metabolism. Knocking out the *Neu2* gene in mice abrogated overall lipid metabolism, impairing motor function.

## Materials and methods

### Generation of Neu2 KO mice

The single guide RNA (sgRNA) including the sequence targeting the Neu2 gene to delete the Neu2 gene was synthesized by ToolGen Inc. (Seoul, Korea), and the CRISPR/Cas9 system was delivered to zygotes obtained from pregnant female C57BL/6J mice and microinjected into female ICR mice. After heterozygous mice (hetero, Neu2^+/−^) were obtained, homozygous Neu2 KO mice (Neu2 KO, Neu2^−/−^) were generated by mating heterozygous littermates to minimize analytical error. The genotypes of the Neu2 KO mice were analyzed by genotyping the offspring using T7 endonuclease 1 (T7E1) analysis of the genomic DNA obtained from mouse tail-tip and were confirmed by Sanger sequencing. We selected Neu2 KO mice with a 14-base deletion in the Neu2 sequence. All use and maintenance processes involving mice were conducted under review and approval of the Institutional Animal Care and Use Committee (IACUC) of KRIBB; this study was reported in accordance with the ARRIVE guidelines. All mice were maintained in a pathogen-free facility at 24 °C, humidity of 40%, and a light–dark cycle of 12 h. Experiments were conducted using littermates unless otherwise stated. Where necessary, experimental groups included multiple litters for statistical power. All experiments were performed in accordance with the relevant guide lines and regulations.

### Histochemistry

Paraffin microsections were cut at a thickness of 4 µm, deparaffinized in xylene, and rehydrated in graded alcohol (Merck). For immunostaining, the sections were subjected to heat-induced epitope retrieval with citrate buffer (pH 6.0) for 3 min at 121 °C. The endogenous peroxidase activity was blocked with 0.3% hydrogen peroxide for 10 min at room temperature, followed by washing in PBS buffer. The sections were treated with serum-free blocking solution (Dako, Glostrup, Denmark) for 20 min at room temperature to block nonspecific binding and then incubated with the indicated antibody against Neu2 (Biorbyt, Cambridge, UK), OXPAT (Novus Biologicals), or MyoD (Novus Biologicals, Centennial, CO, USA) or incubated with Sambucus Nigra Bark Lectin (Vector Labs, Burlingame, CA, USA) overnight at 4 °C. After washing, the sections were incubated for 30 min at room temperature with HRP-labeled polymer-conjugated secondary antibodies against rabbit IgG or mouse IgG and then counterstained with Mayer’s hematoxylin (Sigma-Aldrich) for 30 s before dehydration and mounting with Permount (Thermo Fisher Scientific). For liver fibrosis assessment, the paraffin sections were stained using a Trichrome Stain Kit (StatLab) according to the manufacturer’s instructions. Virtual slides were created by scanning of all stained samples with a ScanScopeAT (Leica Biosystems, CA, USA). All histochemical analysis was performed on a minimum of five mice per group, and random quantification was performed.

### Biochemical analysis of blood parameters

Heparin-anticoagulated serum of each mouse was obtained after clotting for 15 min and then centrifuged at 1000×*g* for 10 min at 4 °C. Total protein, albumin, globulin, total bilirubin, aspartate aminotransferase (AST), alanine aminotransferase (ALT), total cholesterol, triglycerides (TG), high-density lipoprotein cholesterol (HDL-C), low-density lipoprotein cholesterol (LDL-C), blood urea nitrogen (BUN), creatinine, and glucose were analyzed by an AU680 Clinical Chemistry Analyzer (Beckman Coulter, Bedford, MA, USA) at DK Korea (Seoul, Korea). All reagents (Sekisui, Tokyo, Japan) and procedures were performed according to the manufacturer’s protocols.

### Free fatty acid analysis

The quickly frozen tissues were homogenized in 10 volumes of tissue lysis buffer [25 mM Tris–HCl pH 7.4, 150 mM NaCl, 1% NP-40, 1% Na-deoxycholate, 0.1% sodium dodecyl sulfate (SDS), 1 mM Na3VO4, 10 mM NaF, 0.4 mM PMSF, 1 × Protease inhibitor cocktail (Roche, Basel, Switzerland), and 1 × phosphatase inhibitors (Roche)] using a mortar and pestle with liquid nitrogen. The homogenized samples were centrifuged at 13,000*g* for 10 min at 4 °C, and the supernatants were aliquoted and stored at − 80 °C until use. The proteins were quantified with an EZ-Free Fatty Acid Assay Kit (DoGenBio, Seoul, Korea) according to the manufacturer’s instructions.

### Rotarod test

All tests took place during the light phase of the light and dark cycle. The motor coordination and balance ability of the WT and Neu2 KO mice were examined on a rotarod apparatus (BS Technolab, Korea) as previously described^8^, and the body weight of the mice was measured. The day before the test, the mice were acclimated by running a similar experimental protocol on the same machine. The test was repeated three times a day for five consecutive days. The rotarod machine was programmed to automatically record the latency of the mice to fall off the rod with a sensor. The rod was rotated at an acceleration speed from 4 to 50 rpm for 5 min and then continuously at 50 rpm.

### Measurement of energy expenditure

To evaluate respiratory metabolism, energy expenditure (EE) was calculated after measuring oxygen consumption (VO2) and carbon dioxide production (VCO2) using the OxyletPro System-Physiocage (Panlab, Harvard Apparatus, Holliston, MA, USA) for five days in individually caged animals. The measurement value was recorded continuously. VO2 and VCO2 were recorded every 20 min, and the RQ ratio was calculated by dividing VCO2 by VO2. The EE was corrected by a ratio of 0.75 squared by body weight.

### Measurement of body weight

The body weight of the WT control and Neu2 KO mice was monitored for 10 weeks from weeks 6 to 16 in at least 10 mice per group per week.

### Preparation of mouse blood and tissue samples

Mice were fasted for at least 12 h before being sacrificed after anesthesia with intraperitoneal injection of a 1.2% avertin (2, 2, 2,-Tribromoethanol, Sigma-Aldrich) solution to each mouse at a dosage of 240 mg/kg per bodyweight. Mouse blood was obtained from the retro-orbital sinus into heparinized micro-hematocrit capillary tubes (VWR, Wayne, PA, USA) in deeply anesthetized mice and was analyzed within four hours. For protein analyses, each mouse tissue was quickly frozen by liquid nitrogen immediately after dissection and was preserved at − 80 °C until use. For immunohistochemistry analysis, each extracted tissue was fixed in 10% buffered formalin for 24 h, and a paraffin block was prepared and used for histological analyses.

### Glycopeptide preparation and LC–MS/MS analysis

Frozen tissue samples from male WT control and Neu2 KO mice were prepared for proteome and glycopeptide analyses as described by Lee et al.^9^. The samples homogenized with Tris-lysis buffer were sonicated for 1 min and centrifuged for 30 min at 13,000*g* and 4 °C. Measurement of the protein content was performed with the Qubit assay. Protein samples (100 μg) were reduced, alkylated, and digested by Trypsin at 37 °C overnight. According to the manufacturer’s protocols with minor modification, HILIC enrichment was performed for analysis of glycopeptides with the prepared peptide samples^10^. LC–MS/MS analysis was performed with an LTQ-Orbitrap mass spectrometer (Fusion LUMOS, Thermo Fisher Scientific) equipped with an EASY-nLC system (Thermo Fisher Scientific) in high-energy collisional dissociation (HCD)-only mode or HCD product-dependent mode, followed by collision-induced dissociation (CID) for proteome or glycoproteome analysis, respectively^10,11^. The HCD product-dependent mode involved two or more of the five oxonium ions: 126.0550, 138.0555, 168.0661, 186.0766, and 204.0872.

### Identification and quantification of glycopeptides

The raw files from LC–MS/MS were converted into ms1 and ms2 files with RawConverter (Ver 1.1.0.18, The Scripps Research Institute) with the option of data-dependent mode and selection of monoisotopic m/z^11^. To generate a glycopeptide database, proteomic analysis was performed using the Integrated Proteomics Pipeline (IP2, version 5.1.2, Integrated Proteomics Applications Inc., San Diego, CA) and ProteinInferencer (version 1.0, The Scripps Research Institute) with the Uniprot mouse database (download at Jan 2020) and 1% FDR at spectrum and protein levels^10,11^. Glycopeptide analysis was performed with the Integrated GlycoProteome Analyzer (I-GPA; http://iqgpa.org), where the glycopeptide databases in question were generated with the proteins identified in each sample and a combination of 398 and 17 N-glycans and O-glycans^9,11^. To identify protein candidates with glycopeptides discriminating between the WT control and Neu2 KO mice, we performed a T-test assessing the means of two independent samples of glycoprotein intensities using an in-house program (coded by Python 3.8) and the ttest_ind module from SciPy Stats (https://docs.scipy.org/doc/scipy/index.html). The steps for assessing an individual protein were as follows. (1) For comparing all glycoprotein intensities in each sample, glycoprotein intensity was calculated as the sum of the identified glycopeptide intensities in each of protein filtered by their corresponding retention time (< 1.5 min) and the relative intensity of sialic acid peaks (signal to noise > 3.0; 292.1032 and 274.0927). (2) The glycoprotein intensity data table was analyzed by Levene and T-test using the SciPy Stats module, where protein candidates with a p value < 0.05 were extracted via two tandem analyses. (3) A heat map was drawn using the in-house program via the clustermap function of the Seaborn module of Python 3.8 (https://seaborn.pydata.org/generated/seaborn.clustermap.html) using Euclidean metric calculation.

### Intensity quantification of OXPAT

To quantitate OXPAT staining in the liver tissue, 10 fields of a 1 × 10^6^ µm^2^ area per sample were randomly selected from the elderly mice. DAB staining intensity corresponding to OXPAT expression was calculated using the positive pixel counting algorithm of ImageScope software (Leica Biosystems) on selected images.

### Area quantification of vesicles

To evaluate hepatic steatosis, H&E-stained liver tissue was randomly acquired from six fields (4052 × 3028 pixels per field) with a 20X objective lens (0.5 micron/pixel) of a Vectra3 (PerkinElmer, Waltham, MA, USA) multispectral imaging system. Each chromogen has unique spectral characteristics that are used to separate the signals on the image based on a spectral curve. The unique spectral curve corresponding to eosin was used to separate the signals on the acquired images for quantitation. The trainable tissue segmentation algorithm of the InForm (PerkinElmer) software was used to segment hepatic steatosis compartments. Then, the area within the selected compartments of interest was quantitated.

### Area quantification of fibrosis

The degree of fibrosis was measured from digitized images of Masson’s trichrome (MT)-stained liver sections of elderly mice. In the scanned images, 10 fields of a 1 × 10^6^ µm^2^ area per sample were randomly selected. The specific blue pixel areas corresponding to MT were quantified using a positive pixel counting algorithm.

### Area quantification of the myofiber and perimysium areas

To assess the myocyte and perimysium areas in the EDL muscle, two regions of 2 × 10^5^ µm^2^ per sample were randomly selected from the H&E-stained EDL tissue. The muscle area corresponding to the pink pixel in the selected image was quantified using the Imagescope program. The number of myocytes was manually counted in the selected region. The average size of the myocytes was quantified by dividing the muscle area by the number of myocytes, and the perimysium area was quantified by subtracting the area of the muscle from the selected region area.

### Statistical analysis

Results are presented as the mean ± SEM of independent experiments. Significant differences between the two variables in the evaluation of rotarod exercise capacity were analyzed using the Generalized Estimating Equation (GEE) of SAS version 9.4 software (SAS Institute Inc). Statistically significant differences in all other data were analyzed using Student's t-test. The following p-values were considered to be statistically significant: p value ≤ 0.05 (*), p value ≤ 0.01 (**), and p value ≤ 0.001(***).

## Results

### *Neu2-*Deficiency in vivo results in lipid accumulation and a fatty liver

**Figure 1:**
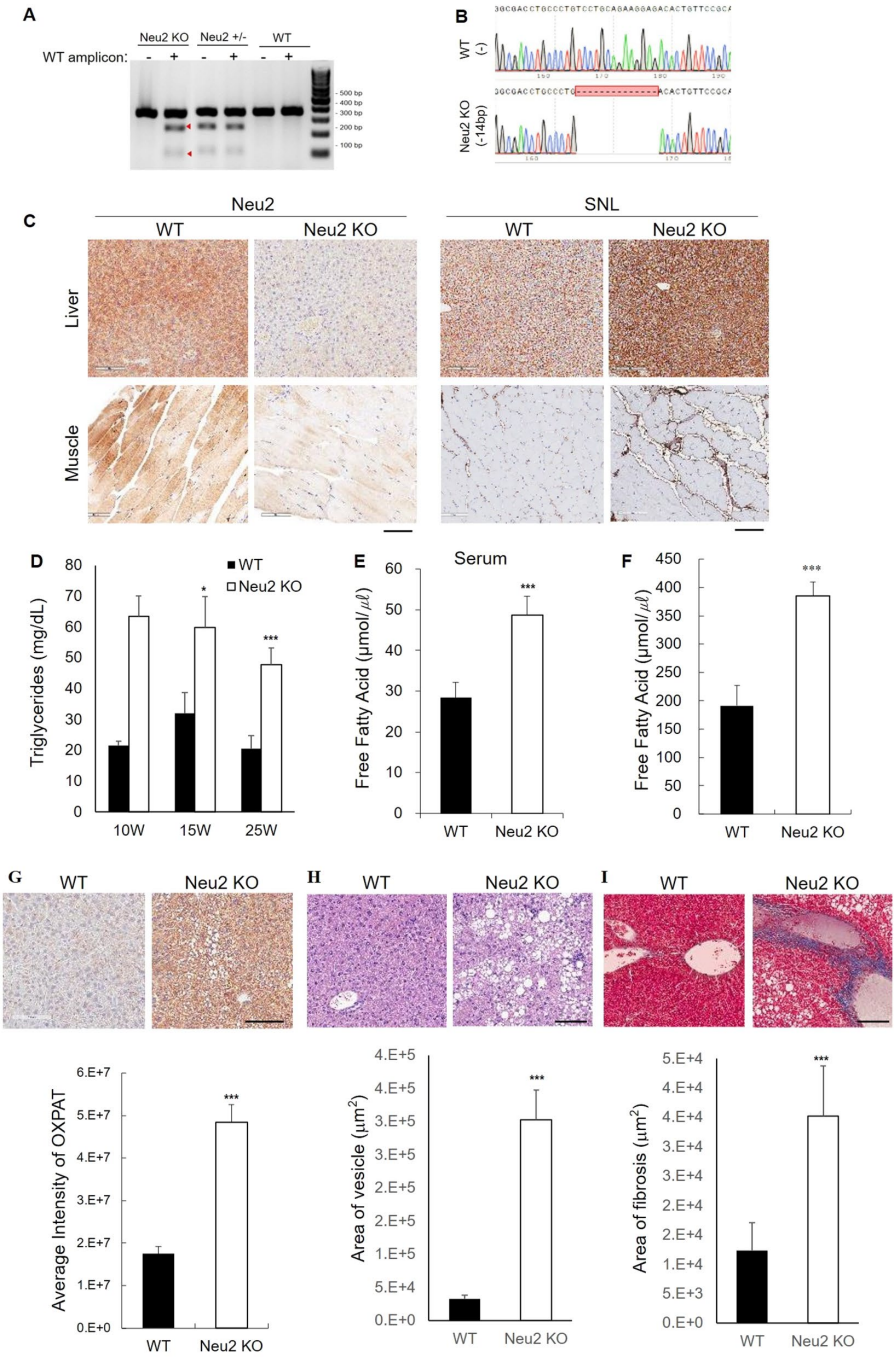
Characterization of the Neu2 KO mouse model. (**A**) Confirmation of *Neu2* gene KO in vivo by T7E1 digestion assay. PCR amplicons of the WT *Neu2* sequence were added to discriminate the homozygous *Neu2* KO from WT. Digested bands are marked by red arrowheads. (**B**) Validation of homozygous *Neu2* gene KO by Sanger sequencing. Deleted bases in the KO mouse are marked by a red block with dashes. (**C**) Histological localization of Neu2 and sialylated glycoproteins by Neu2 immunostaining and SNL staining on paraffin-embedded slides of liver and muscle from adult male mice. Scale bar: 100 μm. (**D**) Biochemical analysis of triglyceride content in serum samples from littermates in the young and adult mice. Young (9–10 w); WT: n = 6; Neu2 KO: n = 7, adult (23–25 w) ; WT: n = 6; Neu2 KO: n = 5. (**E**,**F**) Biochemical quantification of the free fatty acid (FFA) content in adult (23–25 w) serum and elderly (41–55 w) liver samples from littermates. (**E**) Serum FFA content from adult mice; WT: n = 5; Neu2 KO: n = 5, (**F**) FFA content from liver tissues of the elderly (41–55 w) mice. WT (n = 7); Neu2 KO (n = 5). (**G**) Representative lipid droplet marker OXPAT immunostaining and quantification in the liver from elderly (41–55 w) male mice. WT (n = 4); Neu2 KO (n = 4). Scale bar: 100 μm. (**H**) Representative H&E images and quantification of steatosis in the liver from elderly (41–55 w) male mice. WT (n = 4); Neu2 KO (n = 4). Scale bar: 100 μm. (**I**) Representative images and quantification of liver fibrosis from elderly (41–55 w) male mice. WT(n = 4); Neu2 KO (n = 4). Scale bar: 100 μm. All studies were conducted in control and Neu2 KO mice among young (9–10 w), adult (23–25 w), and elderly (41–55 w) male littermates, unless otherwise indicated. Data are expressed as the mean ± SEM. *p < 0.05; **p < 0.01; ***p < 0.0001 by Student’s t-test.

We generated heterozygous C57BL6LJ mice (Neu2^+/-^) through deletion of the *Neu2* gene using the CRISPR/Cas9 system to explore the biological function of *Neu2 *in vivo. Next, homozygous Neu2 KO (N2KO) mice were generated by mating heterozygous mice (Figure S1A and Figure S1B). The genotypes of the Neu2 KO mice were analyzed by genotyping the offspring using T7 endonuclease 1 (T7E1) analysis of the genomic DNA obtained from the mouse tail-tip (Fig. 1A) and confirmed by Sanger sequencing. We confirmed a 14-base deletion in the Neu2 KO mice (Fig. 1B). To further confirm the consequences of the *Neu2* gene knockout, the absence of Neu2 protein expression in homozygous Neu2 KO mice was analyzed by immunohistochemistry in selected mouse tissues including liver, muscle, and brain. Neu2 protein expression was not detected in the Neu2 KO mice tissues examined (Fig. 1C and Figure S1C). Because Neu2 sialidase deficiency was expected to increase the sialylated glycoconjugates in the cytoplasm, we assessed Neu2 sialidase deficiency by staining tissues obtained from the Neu2 KO mice and their control wild-type (WT) littermates with *Sambucus nigra* lectin (SNL). Consistent with the *Neu2* gene knockout, the amount of SNL-bound sialylated glycoconjugates was increased in the liver and muscle connective tissue in the *extensor digitorum longus* (EDL) muscles of the Neu2 KO mice compared to those of the control WT littermates (Fig. 1C). After confirming *Neu2* gene knockout in mice, we analyzed the impact of Neu2 sialidase deficiency by serum biochemical analysis with age. To our surprise, the concentration of serum triglycerides (TG) was significantly higher in the age-matched Neu2 KO mice at 9–10 weeks compared to those in the control WT mice (Table S1). The levels of blood total cholesterol and BUN also were significantly elevated. Further analyses of the littermates revealed that the concentrations of TG in the Neu2 KO mice increased significantly in the young (9–10 w) and adult (23–25 w) age groups compared with their control WT littermates, respectively (Fig. 1D). Because an elevated TG level can lead to an increase in free fatty acid (FFA), the FFA level was measured in these mice. As expected, the serum FFA concentration in the adult group increased 1.7-fold in the Neu2 KO mice compared with that in their control WT littermates (Fig. 1E). Serum hyperlipidemia often is associated with fatty liver disease^12,13^. Hence, we next measured the FFA level in the liver tissue lysates in the elderly (41–55 w) age group. The FFA level increased by 1.9-fold in the Neu2 KO mice livers compared to that in their control WT littermates (Fig. 1F). Because elevated FFA in the liver can contribute to liver steatosis^12,13^, we analyzed whether *Neu2* gene knockout induced the accumulation of lipids in the liver. Using OXPAT as a lipid droplet marker, we assessed the effects of Neu2 gene knockout in vivo and observed marked increases of lipid droplets and lipid accumulation in the liver of the Neu2 KO mice compared with those in their control WT littermates in the elderly age group (Fig. 1G). Excess elevation of blood FFA can cause lipid accumulation in the liver, which can cause inflammatory processes and a fatty liver, leading to fibrous liver scarring around the liver blood vessels^13^. To explore this possibility, we assessed liver steatosis and observed severe steatosis in the livers of elderly Neu2 KO mice (> 41 weeks) (Fig. 1H). We further detected liver fibrosis by staining with Masson’s trichrome (MT) in areas of hepatic steatosis (Fig. 1I). However, hepatic steatosis and fibrosis were not observed in the Neu2 KO mice at 25 weeks or earlier (Figure S1D-1E). Taken together, these results demonstrate that *Neu2* gene deficiency dysregulates lipid metabolism in the liver by increasing the serum TG and FFA levels starting at 9 weeks of age and accumulation of lipid droplets in the liver, finally leading to fatty liver lesions in the elderly Neu2 KO mice (> 41 weeks of age).

### Neu2 sialidase defects impair muscle differentiation and alter muscle morphology

**Figure 2:**
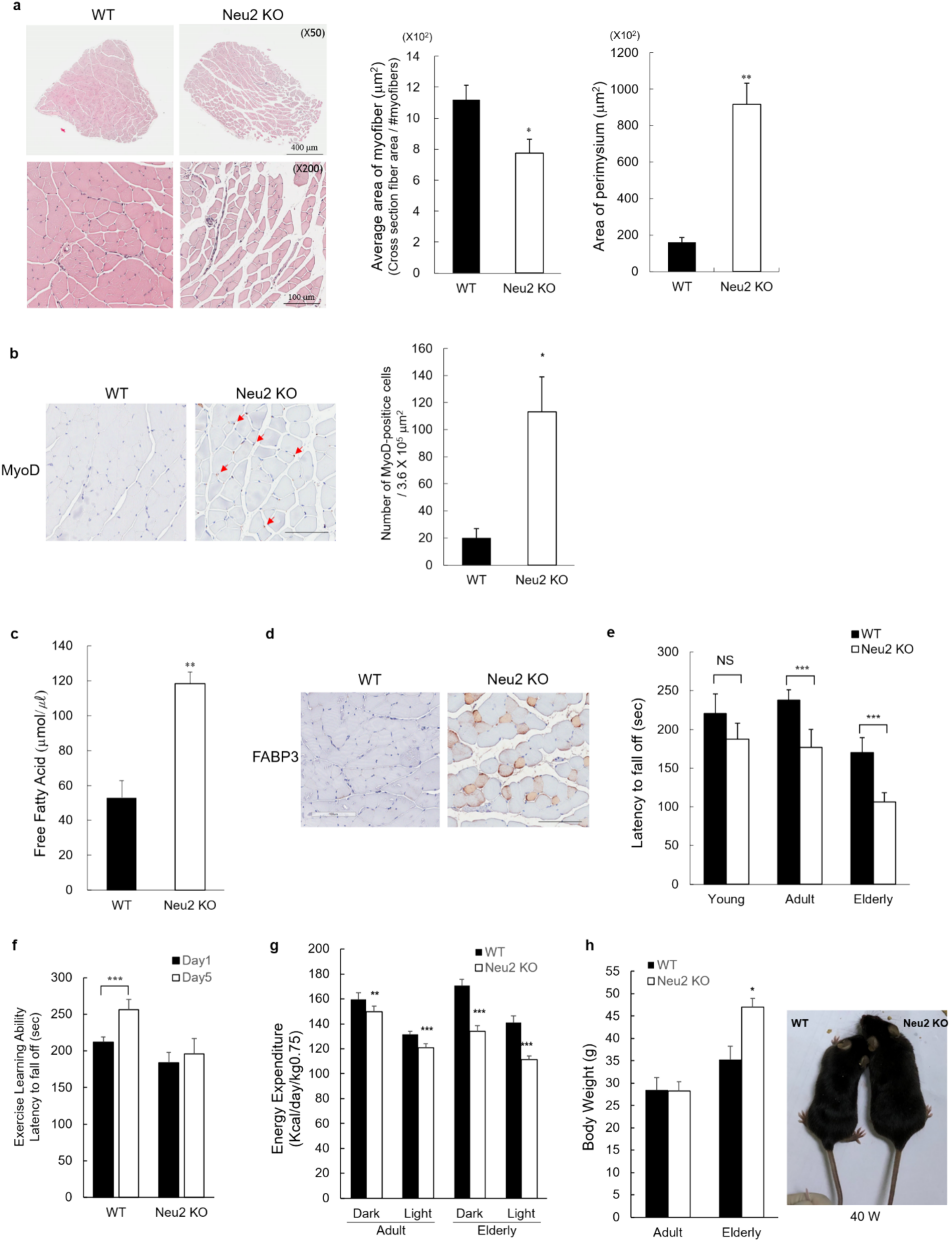
Neu2 deficiency affects muscle fiber morphology and muscle performance. (**a**) H&E staining and quantitation of myofibers and perimysium areas in cross sections of EDL muscle from adult mice. WT (n = 5); Neu2 KO (n = 5). Scale bar: upper panel, 400 μm; lower panel, 100 μm. (**b**) Cross-sections of EDL muscle from adult mice were stained for muscle differentiation using antibodies against MyoD and quantified. WT (n = 5); Neu2 KO (n = 5). Scale bar: 100 μm. (**c**,**d**) Measurement of FFA (**c**) and immunochemistry of FABP3 (**d**) in the EDL muscle of WT and Neu2 KO from elderly mice. WT (n = 4); Neu2 KO (n = 4). Scale bar: 100 μm. (**e**) Latency times from individual littermate performances on the rotarod test. Young, WT: n = 10 and Neu2 KO: n = 9; adult, WT: n = 9; Neu2 KO: n = 9; Elderly, WT: n = 5; Neu2 KO: n = 3. The statistical analysis was performed using the Generalized Estimating Equation (GEE) method, and significance was confirmed at a significance level less than 5%. NS (no significance); ***p < 0.0001. (**f**) Exercise learning ability in the rotarod exercise trials was measured using the results of five trials with adult mice. WT: n = 12; Neu2 KO: n = 15. (**g**) Decrease in energy expenditure by Neu2 deficiency. Energy expenditure was calculated from the adult and elderly mice using the calorimetric parameters obtained during the light and dark phases and formula described in the Methods. Adult; WT: n = 3; Neu2 KO: n = 3, elderly; WT: n = 4; Neu2 KO: n = 3. (**h**) Increases in body weight and obese phenotypic appearance in *Neu2*-deficient elderly mice. Adult; WT: n = 6; Neu2 KO: n = 5, elderly; WT: n = 7; Neu2 KO: n = 7. All studies were conducted in control and Neu2 KO mice among young (9–10 w), adult (23–25 w), and elderly (41–55 w) male littermates, unless otherwise indicated. Data are expressed as the mean ± SEM. *p < 0.05; **p < 0.01; **p < 0.0001 significance confirmed by Student’s t-test, unless otherwise indicated.

Skeletal muscles are an important player in lipid metabolism, and intracellular accumulation of FFA affects both muscle mass and function^14^. Because *Neu2* gene deficiency causes a dysfunction in lipid metabolism by increasing the TG and FFA levels (Fig. 1), it is possible that alterations to the lipid metabolism by the *Neu2* deficiency lead to impairments in muscle morphology and function. To address this possibility, we first evaluated the effect of the *Neu2* gene deficiency in muscle morphology by histological analysis. The EDL muscle is a fast-twitch muscle that is rich in type II glycolytic fibers and thereby relies primarily on anaerobic metabolism to sustain a short, high-intensity activity^15^. Thus, the EDL muscle was used for further analyses. We conducted H&E staining of the EDL muscles to examine the muscle fibers and perimysium in samples obtained from 25-week-old Neu2 KO mice and their control WT littermates to analyze the effects of the *Neu2* deficiency in the Neu2 KO mice muscle tissue. Macroscopic observation of the H&E-stained EDL muscle from the Neu2 KO mice showed a marked difference in the muscle fibers and perimysium compared to the WT controls (Fig. 2a). A marked decrease in EDL muscle myofibers and a striking increase in the area of the perimysium in the EDL muscle of the Neu2 KO mice were evident compared with their control WT littermates. Quantification analysis showed a 30% decrease in EDL muscle myofiber and a greater than 5.7-fold increase in the perimysium in the Neu2 KO mice compared with their control WT littermates (Fig. 2a). Next, we carried out immunohistochemistry with the MyoD antibody to assess the effect of the *Neu2* deficiency in muscle differentiation. Based on the well-established role of the *Neu2* gene in muscle differentiation in vitro, we expected to observe impaired muscle differentiation in the Neu2 KO mice. Immunohistochemistry showed a marked increase (5.6-fold) in the number of nuclear MyoD-positive cells in the EDL muscles of 25-week-old Neu2 KO mice compared with the WT controls (Fig. 2b). During myogenic lineage differentiation, the number of MyoD-expressing cells peaked at the myoblast stage and gradually decreased upon muscle differentiation and maturation^16^. This result suggests that muscle differentiation was affected by the Neu2 deficiency, as reported by previous investigators^17–19^.

Next, to investigate the underlying mechanism of this alteration of muscle morphology and defect in muscle differentiation by the *Neu2* deficiency, we examined the excess accumulation of lipids in the EDL muscle. We observed an increase in FFA in the EDL muscle lysates of the Neu2 KO mice compared with the WT controls (Fig. 2c). According to a recent report, excess plasma FFA induced the accumulation of long-chain fatty acids in muscles through lipid transporters and impaired uptake of glucose in the muscles^20^. Thus, the same muscles were analyzed for the presence of lipid droplets using the fatty acid binding protein 3 (FABP3) antibody by immunohistochemistry. As anticipated, strong FABP3 staining was observed in the EDL muscle of the Neu2 KO mouse (Fig. 2d).

### Neu2 deficiency results in poor exercise performance and induces obesity

Because *Neu2* deficiency resulted in lipid accumulation in the circulating blood, liver, and muscles of the Neu2 KO mice, we investigated whether the lack of *Neu2* gene *in viv*o also led to impairment in muscle performance. To address this question, Neu2 KO and WT littermates were subjected to rotarod and treadmill exercise tests. The rotarod test is used to evaluate motor coordination and motor learning ability by measuring the time until fall from a spinning rod as the speed increases. Here, we used the littermate mice of the young, adult, and elderly groups. Interestingly, knockout of the *Neu2* gene led to a decrease in rotarod exercise performance among the adult and elderly groups of test mice compared with the WT littermates. The motor performance of the Neu2 KO mice was decreased by 42.4 s in the adult group and 69.3 s in the elderly group compared to their corresponding control WT littermates (Fig. 2e). Rotarod motor performance in the Neu2 KO mice significantly decreased with increasing age with maximum deficiency in the elderly group.

Next, to analyze the motor learning ability according to *Neu2* gene deficiency, control WT and Neu2 KO littermate mice of the adult group were subjected to forced rotarod exercise three times daily for five consecutive days. The WT mice significantly improved in motor learning ability over the five consecutive days, and their performance peaked at day 5 (Fig. 2f). In contrast, the motor learning ability of the Neu2 KO mice during consecutive experimental days did not significantly improve compared to that of the control WT littermates. These results suggest that *Neu2* gene knockout affects the exercise learning ability.

Next, we performed the treadmill test, which requires greater endurance than the rotarod test. The treadmill exercise performance test was conducted with mice in adult and elderly age groups. The distance traveled in the treadmill exercise was slightly shorter in the Neu2 KO mice compared to the control WT littermates in both groups (Figure S2A). Because energy production is important for exercise performance, we performed a calorimetric analysis of the littermate mice in the adult and elderly groups to determine the effect of Neu2 knockout on energy production. The Neu2 KO and WT littermates were analyzed by measuring oxygen consumption (VO_2_) and carbon dioxide production (VCO_2_) for 5 days at the resting condition. Calorimetric measurements revealed low oxygen consumption and carbon dioxide production in the Neu2 KO mice in the elderly age group (Figure S2B and S2C). Calculation of the energy expenditure using VO_2_ and VCO_2_ showed a lower energy expenditure in Neu2 KO mice in both the adult and elderly groups compared to those in their WT littermates during both day and night (Fig. 2g). The energy expenditure rate of the Neu2 KO mice was reduced in the adult group and further reduced in the elderly group compared to that in the control WT mice. Noticeably, the energy consumption of elderly mice in the dark condition decreased by up to 21% in the Neu2 KO mice compared to the control WT littermates, suggesting that *Neu2* gene deficiency in mice seriously affects energy consumption in vivo. Because energy expenditure is related to obesity, we monitored body weight from 6 to 25 weeks of age in the Neu2 KO and WT littermate mice. The body weights of the Neu2KO mice gradually changed over 10 weeks from weeks 6 to 16 (Figure S2D). In the elderly group, noticeably, an obese phenotype and a significant body weight increase (> 1.3-fold) were observed in the Neu2 KO mice compared with their control WT littermates (Fig. 2h).

### Neu2 deficiency leads to alterations of sialylated glycoproteins involved in the regulation of both lipid metabolism and muscle function

**Figure 3:**
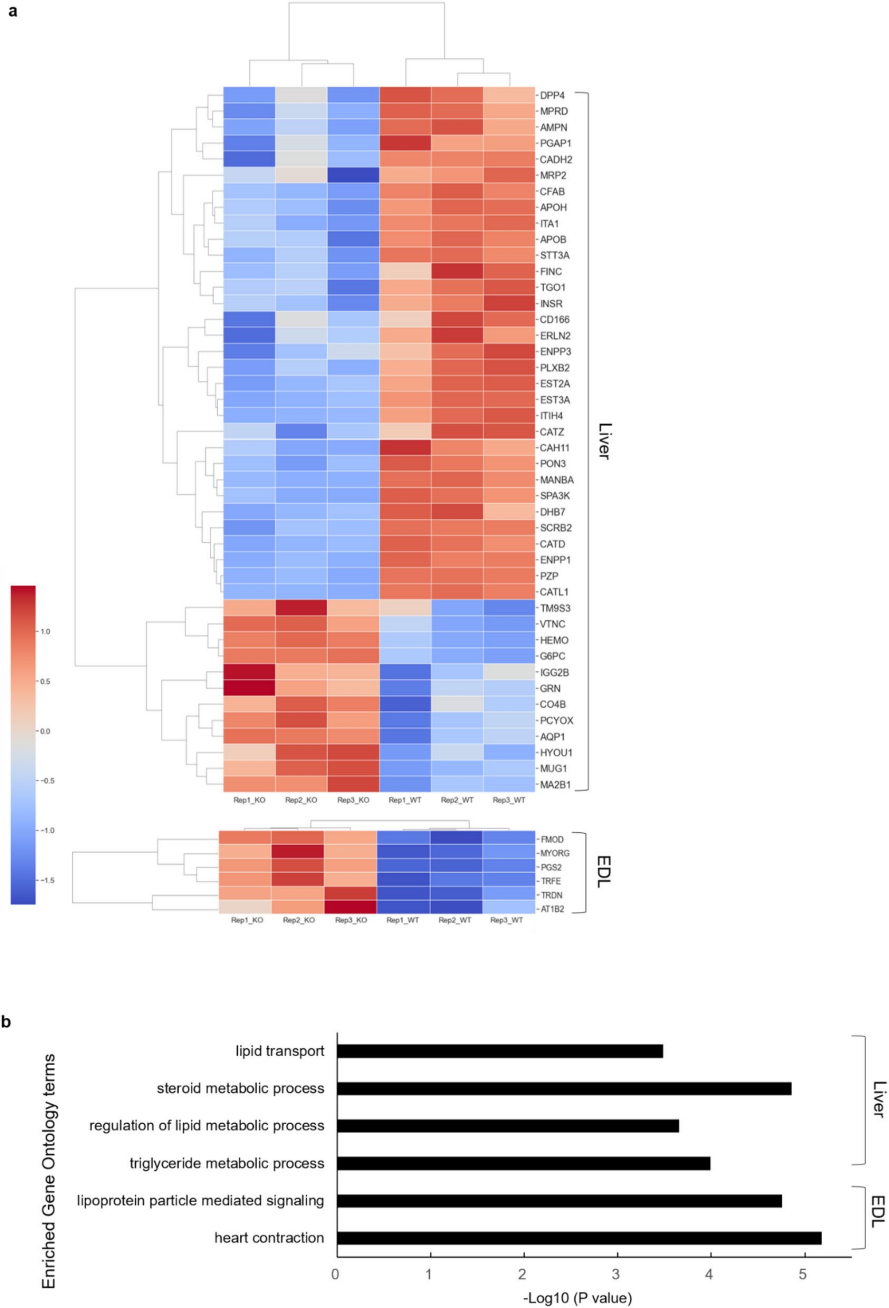
*Neu2* Deficiency affects sialylated glycoproteins regulating lipid metabolism and muscle function. (**a**) A heat map of the identified sialylated glycoproteins in the liver and EDL muscle. Glycoprotein intensity analysis and T-test were performed using an in-house program coded by Python 3.8 with the SciPy Stats module, while the heat map was drawn using the Seaborn Clustermap module. (**b**) Biological process enrichment analysis showed identification of sialylated glycoproteins involved in lipid metabolism and muscle functionality. All studies were conducted in control and Neu2 KO littermates 25 weeks of age. WT: n = 1; Neu2 KO: n = 1. Data are obtained in triplicate experiments.

We identified target proteins involved in the regulation of lipid metabolism and muscle functions to elucidate the underlying mechanism leading to abrogation of lipid metabolism by *Neu2* deficiency. We identified sialylated glycoproteins through glycoproteomics analysis with high-resolution tandem mass spectrometry (Fig. 3a,b). Of the 2,103 proteins identified using the Integrated Proteomics Pipeline (IP2), 234 glycoproteins with one or more glycosylation sites according to the Uniprot database (January 2019, http://www.uniprot.org) were selected for glycoproteomics analysis. A total of 68 glycoproteins was identified with or without SA from the liver tissues of the Neu2 KO and control WT littermates. Here, 12 and 32 glycoproteins showed an increase and decrease, respectively, in the Neu2 KO samples. The increases and decreases of glycoproteins showed different patterns seen in triplicate (Fig. 3a). Gene ontology analysis using the Panther Classification System (http://geneontology.org) revealed that lipid metabolism-related gene ontology (GO) terms were enriched in the presence of 36 glycoproteins identified in biological processes (Fig. 3b). We identified four N-glycopeptides having one or more SA molecules: apolipoprotein B (ApoB), transport and Golgi organization protein 1 homolog (TANGO1), paraoxonase1 (Pon1), and 3-keto-steroid reductase (DHB7) (Table 1). The glycan structure of 5_4_0_0_2 (HexNAc_Hex_Fuc_NeuAC_NeuGC) was identified attached to N-glycopeptides such as LTYESGFLNYSK (ApoB, 2,982th N) and HANWTLTPLK (PON1, 270th N). Structures 6_4_1_0_1 and 4_3_0_0_2 were identified from ANFSLEDIQHSK (DHB7, 178th N) and DPNLSEEDK (TGO1, 360th N), respectively. All these N-glycopeptides can be classified as complex type N-glycans with NeuGC of SA. Six MS raw files of the WT and Neu2 KO liver tissues with triplicates were deposited in a database (https://www.igpa.kr/), in which all identified glycopeptide spectra are included. Among the glycoproteins analyzed, those showing a quantitative change in overall sialylation were included (Table 1). Interestingly, the lipid metabolism-associated glycoproteins APOB, DHB7, and TANGO1 were identified and are all directly involved in transport and synthesis of lipid components in the liver. In particular, ApoB is a lipoprotein that carries fat molecules including triglycerides and cholesterol. High levels of ApoB are closely related to triglyceride and cholesterol concentrations and are a major cause of plaque resulting in vascular disease (atherosclerosis)^21,22^. In contrast, TANGO1 is a protein that exports bulky lipid particles and interacts with ApoB to export large lipid particles from the ER^23^. Conversely, PON1 is a major anti-atherosclerotic component of high-density lipoprotein (HDL). The pon1 gene is activated by PPAR-γ and increases synthesis and release of paraoxonase I enzyme from the liver, reducing atherosclerosis^24^. In addition, beta-2-glycoprotein 1 (APOH) binds to negatively charged substances including heparin, phospholipids, and dextran sulfate. MRP2 mediates hepatobiliary excretion of numerous organic anions and conjugated organic anions. Lysosome membrane protein 2 (SCRB2) acts as a lysosomal receptor for glucosylceramidase (GBA) targeting. Finally, glucose-6-phosphatase (G6PC) is the key enzyme involved in homeostatic regulation of blood glucose. Namely, G6PC binds with the glucose-6-phosphate transporter and is responsible for glucose production through glycogenolysis and gluconeogenesis.

**Table 1:** Identification of sialylated glycoproteins involved in lipid metabolism and muscle function.

Organ	Gene Symbol	Glycoprotein	Function	Overall sialylation expression pattern
Liver	*Apob*	Apolipoprotein B-100	Apolipoprotein B is a major protein constituent of chylomicrons (apo B-48), LDL (apo B-100) and VLDL (apo B-100). Apo B-100 functions as a recognition signal for the cellular binding and internalization of LDL particles by the apoB/E receptor	Down
	*Hsd17b7*	3-keto-steroid reductase	Bifunctional enzyme involved in steroid-hormone metabolism and cholesterol biosynthesis	Down
	*Mia3*	Transport and Golgi organization protein 1 homolog (TANGO1)	Required for secretion of lipoproteins by participating in their export from the endoplasmic reticulum	Down
	*Pon1*	Serum paraoxonase	Major anti-atherosclerotic component of high-density lipoprotein (HDL). The PON1 gene is activated by PPAR-γ, which increases synthesis and release of paraoxonase 1 enzyme from the liver, reducing atherosclerosis	Down
	*Apoh*	Beta-2-glycoprotein 1	Binds to various kinds of negatively charged substances such as heparin, phospholipids, and dextran sulfate	Down
	*Abcc2*	Canalicular multispecific organic anion transporter 1	Mediates hepatobiliary excretion of numerous organic anions and conjugated organic anions such as methotrexate, 17beta-estradiol 17-glucosiduronic acid, and leukotriene C4	Down
	*Scarb2*	Lysosome membrane protein 2	Acts as a lysosomal receptor for glucosylceramidase (GBA) targeting	Down
	*G6pc*	Glucose-6-phosphatase	Forms with the glucose-6-phosphate transporter (SLC37A4/G6PT). This complex is responsible for glucose production through glycogenolysis and gluconeogenesis. It is the key enzyme in homeostatic regulation of blood glucose level	Up
EDL	*Fmod*	Fibromodulin	Affects the rate of fibril formation. Might have a primary role in collagen fibrillogenesis	Up
	*Myorg*	Myogenesis-regulating glycosidase	Promotes myogenesis by activating AKT signaling through maturation and secretion of IG	Up
	*Dcn*	Decorin	Affects the rate of fibril formation	Up
	*Trdn*	Triadin	Contributes to regulation of luminal Ca^2^ + release via the sarcoplasmic reticulum calcium release channels RYR1 and RYR2, a key step in triggering skeletal and heart muscle contraction	Up
	*Atp1b2*	Sodium/potassium -transporting ATPase subunit beta-2	The non-catalytic component of the active enzyme, which catalyzes the hydrolysis of ATP coupled with the exchange of Na + and K + ions across the plasma membrane	Up
	*Tf*	Serotransferrin	An iron binding transport protein that can bind two Fe^3+^ ions in association with the binding of an anion, usually bicarbonate	Up

To understand the mechanism of *Neu2*-deficiency-mediated muscular dysfunction observed in the Neu2 KO mice, we performed glycopeptide analyses with EDL muscles. Among the sialylated glycoproteins identified, the fibromodulin (Fmod) protein is a main regulator of myostatin, which controls the development of muscle cells, and is abundantly expressed in muscles and connective tissues. The Fmod protein regulates the expression of atrophy-related genes to alleviate muscle atrophy by regulating the TGF signaling pathway^25,26^. In contrast, myogenesis regulating glycosidase (MYORG) is a glycosidase that has an important role in myoblast differentiation and is involved in movement disorders and cognitive impairment^27,28^. On the other hand, PGS2 (decorin) is important for maintenance of glucose tolerance, and PGS2 knockout decreased glucose tolerance^29^. Previous investigations showed that PGS2 acts as a myokine and promotes muscle hypertrophy by binding with myostatin^30^. In contrast, TRDN (triadin) is required for normal skeletal muscle strength and acts in excitation–contraction coupling in the heart^31^. AT1B2 is an ATPase Na + /K + transporting subunit beta 1 that is critical for the electrochemical gradient essential for the electrical excitability of the nerves and muscles. Finally, serotransferrin is an iron-binding transport protein that is regulated in response to exercise^32^. Our glycoproteomics analyses of the liver and EDL muscle tissues led to identification of key sialylated glycoproteins involved in regulation of lipid metabolism and muscle function. These results suggest that Neu2 sialidase deficiency in vivo affects pivotal proteins involved in lipid metabolism and muscle function.

## Discussion

Since the first molecular cloning of mammalian sialidase encoding the NEU2 in 1993^33^, Neu2 sialidase has been recognized as a cytoplasmic enzyme primarily expressed in skeletal muscles^34^, the liver^35^, and the thymus^36^. The best-known role of the *Neu2* gene in the differentiation of skeletal myoblasts was investigated with in vitro studies using overexpression and silencing of the *Neu2* gene in cultured skeletal myoblasts^17,19,37^. There were no previous reports describing the in vivo function of *Neu2*. In the present study, we generated and characterized Neu2 KO mice through phenotypic and functional analyses. To the best of our knowledge, this is the first study reporting the in vivo function of Neu2 sialidase. We observed involvement of the *Neu2* gene in lipid metabolism and muscle function using Neu2 KO mice. Our findings strongly suggest that silencing of the *Neu2* gene abrogates lipid metabolism and consequently impairs muscle morphology and muscle function by regulating key proteins. Our findings provide physiological clues explaining the impairment of muscle function due to abrogation of lipid metabolism, which is key to the regulation of muscle function. Our results support those of recent investigations demonstrating that excess accumulation of lipids impairs insulin sensitivity in skeletal muscles^20^. We also noted striking elevation of FFA and FABP3 in the EDL muscle of Neu2 KO mice compared with control WT littermates in the elderly age group (Fig. 2c,d). Furthermore, we observed a noticeable increase of body weight in the Neu2 KO mice (Fig. 2h), suggesting an association between alteration of lipid metabolism and obesity.

One of the most interesting observations of our analysis of Neu2 knockout mice was the continued decline in rotarod motor performance with age. Our results show that alteration of the muscle fibers and sialylation of key proteins due to *Neu2* gene defects affect exercise performance in mice. *Neu2* gene deficiency led to atrophy in the muscle cells and an increase in the perimysium. Such changes explain the poorer exercise performance of the Neu2 KO mice. Previous investigations demonstrated the role of *Neu2* gene in muscle fiber differentiation in vitro^17,38^. Our findings support the in vitro findings and further demonstrate that *Neu2* deficiency can change the muscle morphology, lipid metabolism, and critical sialylated glycoproteins involved in key biological processes. The decreased exercise capacity in the Neu2 KO mice was anticipated based on previous functional studies in muscle development^39^. While exercise capacity in the treadmill test revealed a slight reduction, we observed significant reductions in the rotarod exercise capacity (Fig. 2e). Unlike the treadmill test, which examines animal endurance and running performance, the rotarod test evaluates balance, grip strength, and motor coordination of the animals tested^40^. Thus, the decreased motor ability in the Neu2 KO mice suggests that Neu2 sialidase defects can affect brain tissues. To address this question, we analyzed the brain tissues of Neu2 KO mice. SNL lectin staining analysis in the Neu2 KO brain tissue showed a marked elevation of sialylated glycoconjugates in the Neu2 KO mouse cerebral cortex and hippocampus compared with the control WT littermates (Figure S1C), suggesting accumulation of sialylated glycoconjugates in those brain regions to consequently affect brain function. Further analysis of glycoproteins in the brain tissues from both the Neu2 KO mouse and control WT littermates revealed upregulation of sialylated glycoproteins involved in regulation of the adhesion of cerebellar neurons, neurite outgrowth and glial cell attachment, neural recognition, and calcium-dependent processes including muscle contraction, neurotransmitter release, and synaptic secretion in neural cells (Supplementary Table S2). Sialylated glycoprotein analysis supported the involvement of Neu2 deficiency in brain-associated diseases such as amyotrophic lateral sclerosis, Alzheimer disease, and Huntington disease. Previous reports have demonstrated that reductions in rat hippocampus sialidase activity resulted in increased surface SA, inhibiting cell adhesion and cellular interactions via synaptic, myelin, and nuclear membranes, consequently leading to pathological changes^41^. According to previous investigations, sialidase has pivotal roles in brain development and neurological disorders^42,43^. *Clostridium perfringens* sialidase^44^ and recombinant *Vibrio cholerae* sialidase^45^ have been proposed as candidate therapeutic agents for spinal cord injury due to their prospective inhibition of glycan binding determinants of myelin-associated glycoproteins, which support enhanced motor and autonomic functional recovery. Thus, a sialidase of human origin is a potential therapeutic option^1^.

One of the remarkable features of the Neu2-deficient mice was dysfunction in lipid metabolism. We observed noticeable changes in blood lipoproteins (such as TG, cholesterol, LDL and HDL) in age-matched Neu2 KO mice at 10 weeks of age. According to previous clinical studies by other investigators, a high level of SA in plasma contributes to increases in cardiovascular disease^46^. The content of the plasma lipoprotein sialic acid (LSA) differs considerably as a result of (1) variation in sialylation of apolipoproteins before secretion into the plasma; (2) variation in the amount of SA-containing apolipoproteins on lipoproteins in the plasma, and (3) modifications of the SA on lipoprotein constituents following secretion into the plasma^47^. The increase in lipoproteins in the serum of the Neu2 KO mice was interpreted as a result of sialylation changes due to Neu2 deletion. These occurred because many sialylation proteins involved in lipid metabolism and synthesis are changed in Neu2 KO mice (as indicated by the sialylated glycopeptide analysis, Table 1). Interestingly, we did not observe significant quantitative changes in sialylation protein mRNA expression by RNA-seq analysis (data not shown). This suggests that the significantly increased lipoproteins in the sera of the Neu2 KO mice were caused by increased sialylation of glycoproteins involved in the regulation of lipid synthesis and degradation. Taken together, our findings provide an important cornerstone for exploring the mechanism of Neu2-mediated altered lipid metabolism in vivo.

The prominent abrogation of lipid metabolism and muscle impairment in Neu2 knockout mice was not observed in the deletion of other sialidases. For example, lysosomal sialidase Neu1-deficient mice showed muscle degeneration caused by infiltration of muscle fibers by expanded connective tissue^48^ and displayed increased proteolytic activity of lysosomal cathepsins and metalloproteinases and lysosomal storage disease (LSD) sialidosis. Furthermore, hearing loss was detected in Neu1-deficient mice by vacuolization and alterations of lysosomal membrane proteins in cochlear marginal cells^49^. In contrast, knockout of the plasma membrane-localized sialidase Neu3 revealed attenuated pulmonary fibrosis^50^. Interestingly, mass spectrometric analysis of the brains of HeXa−/−Neu3−/− mice revealed abnormal accumulation of GM2 ganglioside and progressive neurodegeneration with neuronal loss and skeletal bone abnormalities, mimicking the neuropathological and clinical abnormalities of classical early-onset Tay-Sachs patients in the brain^8^. In contrast, Neu4KO mice exhibited abnormal ganglioside catabolism and lysosomal storage^51^. Especially, a markedly decreased level of GM1 ganglioside was detected in the brain, suggesting that Neu4 is important for desialylation of brain gangliosides. However, we did not observe these characteristics in Neu2 mice. We speculate that differences in subcellular localization, substrate specificity and affinity, and enzyme working pH for the given sialidases might contribute to the phenotypic differences by the respective sialidase deficiency in mice. Further characterization of Neu2 KO mouse tissue other than liver and muscle would provide important information to better characterize the effect of Neu2 deficiency in mice.

In conclusion, we investigated the biological role of cytosolic Neu2 sialidase using a Neu2 KO mouse model (C57BL/6 J) generated by CRISPR/Cas9-mediated genome engineering. The most salient features observed in the Neu2 knockout mice in this study include abrogation of lipid metabolism in the liver and muscles, changes in energy expenditure, and striking body weight gain. We hypothesize that both abrogation of lipid metabolism and impairment of muscle function by *Neu2* deficiency affect the knockout mice throughout life, consequently leading to fatty liver and diabetes. To the best of our knowledge, this is the first study on the biological function of cytosolic Neu2 sialidase. Our findings provide a rationale for use of the Neu2 KO mice as a model of early liver disease and diabetes.

## Supplementary Information

Supplementary Information.
