# Supplementary material for: Defect in cytosolic Neu2 sialidase abrogates lipid metabolism and impairs muscle function in vivo
Source: Sci Rep. 2022 Feb 25;12:3216. doi: 10.1038/s41598-022-07033-6 (PMC8881595; doi:10.1038/s41598-022-07033-6)

**Supplementary Information (Oh et al.)**

**Table S1. (Related to Figure1) Elevation of Triglycerides and total cholesterol in serum of Age-matched Neu2 KO mice**

| **TEST** | **WT** | **Neu2 KO** | | **Neu2 KO/WT (*P*-value)** |
| --- | --- | --- | --- | --- |
| Total Protein (g/dL) | 5.12 ± 0.43 | | 5.97 ± 0.3 | 1.17 (*) |
| Albumin (g/dL) | 2.05 ± 0.13 | | 2.28 ± 0.18 | 1.11 (*) |
| Globulin (g/dL) | 3.07 ± 0.36 | | 3.69 ± 0.23 | 1.20 (*) |
| AST (U/L) | 124.79 ± 19.2 | | 120.16 ± 14.05 | 0.96 |
| ALT (U/L) | 44.91 ± 5.15 | | 58.74 ± 5.96 | 1.31 (*) |
| Total.Cholesterol (mg/dL) | 90.26 ± 4.7 | | 122.16 ± 4.82 | 1.35 (***) |
| TG (mg/dL) | 24.79 ± 4.58 | | 64.98 ± 6.57 | 2.62 (***) |
| HDL-C (mg/dL) | 65.16 ± 4.27 | | 82.33 ± 3.94 | 1.26 (***) |
| LDL-C (mg/dL) | 11.81 ± 1.21 | | 15.35 ± 1.88 | 1.30 (*) |
| BUN (mg/dL) | 18.23 ± 0.88 | | 28.88 ± 2.01 | 1.58 (***) |

Values shown are ± SEM. Statistical analysis shows a significant difference in the indicated parameters. *(*P*<0.05), ***(*P*<0.001).

**Table S2. Identification of sialylated glycoproteins differentially expressed in cerebellum.**

| **Gene Symbol** | **Glycoprotein** | **Function** |  | **Overall sialylation expression pattern** |
| --- | --- | --- | --- | --- |
| *Hspg2* | Basement membrane-specific heparan sulfate proteoglycan core protein | Component of the glomerular basement membrane (GBM), responsible for the fixed negative electrostatic membrane charge, and which provides a barrier which is both size- and charge-selective. It serves as an attachment substrate for cells |  | Down |
| *Gpr149* | Probable G-protein coupled receptor 149 | Orphan receptor |  | Down |
| *Arhgef2* | Rho guanine nucleotide exchange factor 2 | Involved in neuronal progenitor cell division, differentiation, and the migration of precerebellar neurons |  | Down |
| *Rictor* | Rapamycin-insensitive companion of mTOR | Subunit of mTORC2, which regulates cell growth and survival in response to hormonal signals |  | Down |
| *Dusp10* | Dual specificity protein phosphatase 10 | Protein phosphatase involved in the inactivation of MAP kinases |  | Down |
| *Hpx* | Hemopexin | Binds heme and transports it to the liver for breakdown and iron recovery, after which the free hemopexin returns to the circulation |  | Down |
| *Pzp* | Pregnancy zone protein | Can inhibit all four classes of proteinases by a unique 'trapping' mechanism |  | Up |
| *Gpr21* | Probable G-protein coupled receptor 21 | Orphan receptor |  | Up |
| *Dmd* | Dystrophin | Component of the dystrophin-associated glycoprotein complex which accumulates at the neuromuscular junction (NMJ) and at a variety of synapses in the peripheral and central nervous systems, and has a structural function in stabilizing the sarcolemma |  | Up |

**Figure S1. (Related to Figure 1) Generation of the Neu2 KO mice.**

(a) Schematic illustration of the Neu2 KO mouse generation. Some vector icons such as the mouse, cell and DNA were obtained from a free vector icon site 'flaticon' (URL: [https://www.flaticon.com](https://www.google.com/url?q=https%3A%2F%2Fwww.flaticon.com&sa=D&sntz=1&usg=AFQjCNEwPOK_3whfQPg1Wbyg3VnaXMEstQ)). All other components drawn in the figures were produced using the Microsoft Powerpoint. (b) Target design of the Neu2 gene using a CRISPR/Cas9 system. The common exon of all Neu2 isotypes was targeted to knockout the Neu2 protein. NGG PAM sequence (red) and exon regions (grey arrows) are marked, and the DSB site is marked with a red arrowhead. All components drawn in the figure S1b were produced using the Microsoft Powerpoint. (c) Histological expression of the Neu2 protein and sialylated glycoproteins by Neu2 immunostaining and SNL staining on paraffin-embedded slides of adult mouse brain tissue. Scale bar: 100 μm. (d) Representative immunohistochemistry images of OXPAT protein expression in Neu2 KO and WT adult mice. Quantitative analysis of the relative OXPAT protein expression in N2KO and wild-type liver tissues. Scale bar: 100 μm. (e) H&E-stained representative images of liver steatosis of 25-week-old Neu2 KO mice and control WT mice littermates. Scale bar: 100 μm. (f) Representative images of *Masson's trichrome* staining for detection of liver fibrosis in 25-week-old Neu2 KO mice and control WT mice littermates. Scale bar: 100 μm. All studies were conducted in the control and Neu2 KO mice among adult (23-25 w) male littermates, unless otherwise indicated. WT: n = 5; Neu2 KO: n=5. Data are expressed as the mean ± SEM. **p < 0.01 by Student’s t-test.


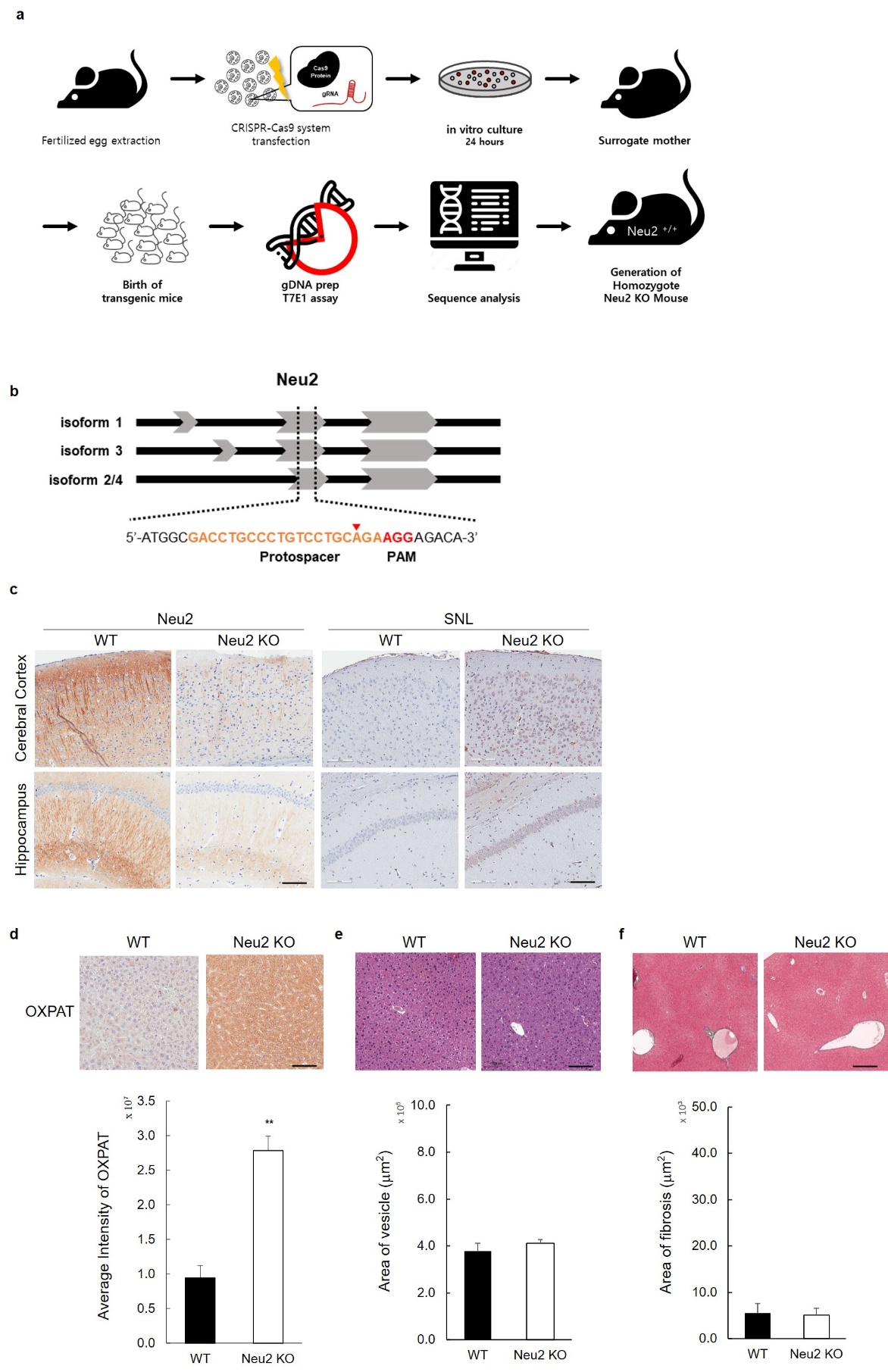


**Figure S2. (Related to Figure 2) Poor muscle performance and obese phenotypic appearance in Neu2 deficient mice.**

(a) Running distance was measured based on individual performance on the treadmill test. Wild-type and Neu2 KO animals in the young (16-20 w) and elderly (43-52 w) age groups were subjected to the treadmill performance test. WT: n = 5, Neu2 KO: n = 3. (b and c) Calorimetric parameters were obtained for the Neu2 KO and control WT mice at 50 weeks of age using metabolic cages. Oxygen consumption and carbon dioxide production were measured during the light phase, dark phase, and overall. WT: n = 4, Neu2 KO; n = 3. (d) Effect of the Neu2 KO on mouse body weight. Body weight was measured from weeks 6 to 16 by direct measurement. WT: n = 5, Neu2 KO: n = 5. Data are expressed as the mean ± SEM. *p < 0.05; **p < 0.01 by Student’s t-test.

**
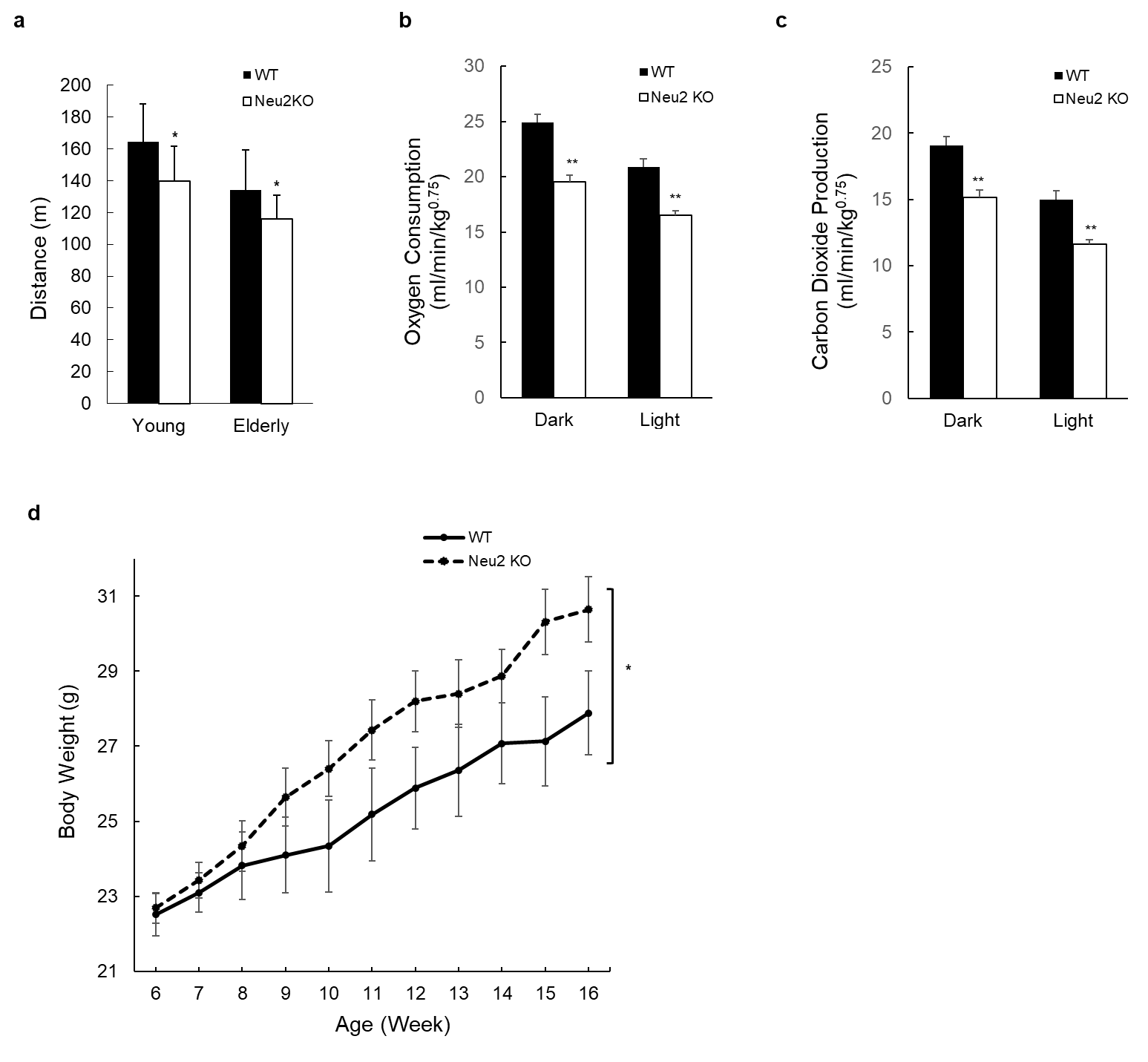
**

**Figure S3. Proteomic and glycoproteomic analyses of sialylated glycoproteins in the liver and EDL muscle.**

(a) An experimental workflow of proteomics and glycoproteomics. The workflow of proteomics and glycoproteomics was drawn using Integrated Proteomic Pipeline (IP2, Bruker) and Integrated GlycoProteome Analyzer (I-GPA; <http://iqgpa.org>), respectively. (b) Tandem mass spectra of CID and HCD identified as LTYESGFLNTSK_5_4_0_0_2 (peptide_HexNac_Hex_Fuc_NeuAC_NeuGC) from ApoB (2,982^nd^ N). The color of the assigned peak indicates oxonium ions, Y ions, and B ions as light blue, red, and blue, respectively. MS/MS spectra image was captured from Integrated Proteomic Pipeline (IP2, Bruker) and Integrated GlycoProteome Analyzer (I-GPA: <http://iggpa.org>].


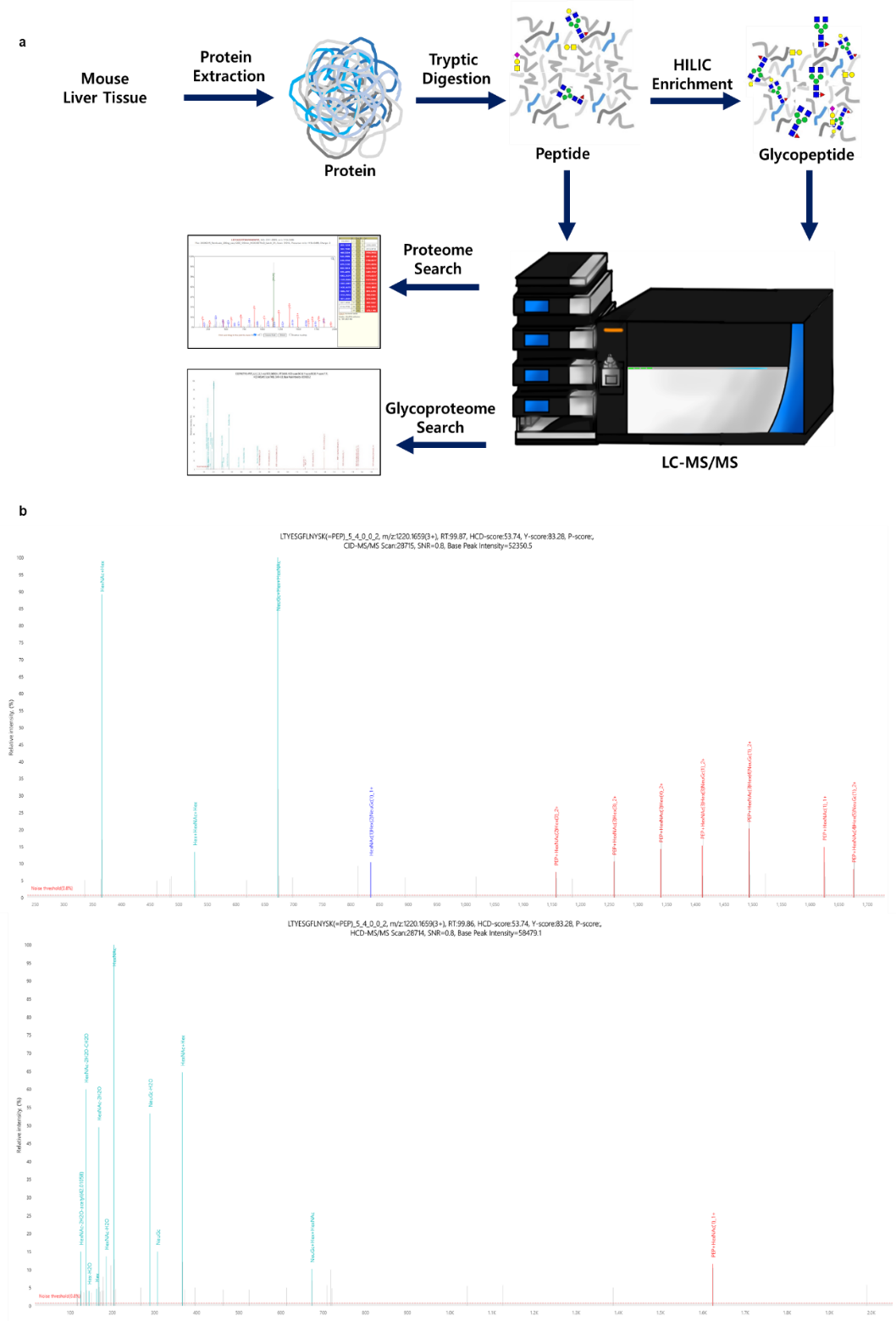


**Figure S4 (Related to Figure 1A). Representative gel image to screen the Neu2 gene knockout *in vivo* by T7E1 digestion assay as described in Materials and Methods.**


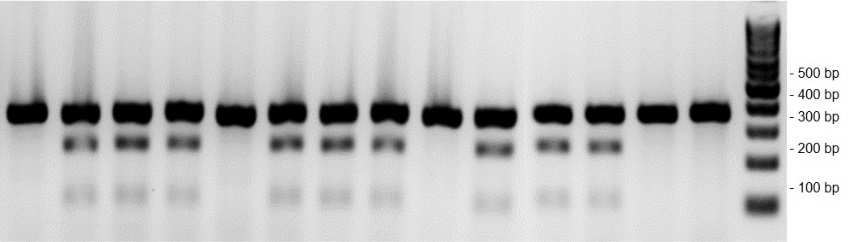

Supplement: Supplementary file 1 — Supplementary Information. [file 41598_2022_7033_MOESM1_ESM.docx]
